# P-1908. The Relationship Between Long COVID and PHQ-4 Measures of Emotional Health

**DOI:** 10.1093/ofid/ofae631.2069

**Published:** 2025-01-29

**Authors:** Christopher Kricorian

**Affiliations:** For Good Measure, Simi Valley, California

## Abstract

**Background:**

The COVID-19 pandemic was one of the most devastating public health crises in US history. Millions of Americans experience ongoing symptoms, often termed “Long COVID.” Long COVID (LC) has been defined as symptoms lasting 3 months or longer that were not present prior to the COVID infection. LC’s neurological effects have been associated with psychological issues like depression. Furthermore, given that many individuals with LC report difficulty getting treatment for these symptoms, the emotional effects associated with LC may be profound.

**Methods:**

The US Census Bureau conducts an ongoing study called the Household Pulse Survey, with the purpose of measuring emergent social and economic matters facing U.S. households. Data analyzed was collected January 9 - February 5, 2024. Respondents were asked about their self-reported COVID-19 experiences and their mental health, as measured by the Patient Health Questionnaire-4 (PHQ-4) for depression and anxiety. The survey was conducted in English and Spanish and the survey sample was N=68,544. Data were analyzed using χ^2^ and between group contrast analysis.

**Results:**

Of those surveyed, N=9,298 or 16.5% reported experiencing LC. When asked about their experiences during the last two weeks, 60.1% reported feeling anxious on several days or more, vs. 40.5% of those who had experienced COVID-19 but had no long term symptoms (termed Short COVID or SC) and 38.7% of those who had not contracted COVID-19 (called No COVID or NC), with LC significantly higher than both other groups (p< .05). Fully 51.1% of LC respondents were unable to stop or control their worrying, vs. 29.1% SC and 31.9% NC= (both comparisons vs. LC, p< .05). Similarly, those with LC were also significantly more likely to report having experienced little pleasure or interest in doing things (47.5% vs. 25.2% SC and 30.4% NC, p< .05) and feeling down, depressed or hopeless in the past two weeks (48.3% vs. 27.4% SC and 30.9% NC, p< .05).

**Conclusion:**

Many Americans surveyed report experiencing LC and this appears to be associated with significant negative mental health effects, as measured by the PHQ-4. Further research should examine the relationship between LC and mental health, as well as mechanisms of how these factors relate to one another.

**Disclosures:**

All Authors: No reported disclosures

